# Mild cognitive impairment: narrative review of taxonomies and systematic review of their prediction of incident Alzheimer's disease dementia

**DOI:** 10.1192/bjb.2019.77

**Published:** 2020-04

**Authors:** Nicholas I. Bradfield, David Ames

**Affiliations:** 1 St Vincent's Hospital Melbourne, Australia; 2 St George's Hospital, Kew, Australia

**Keywords:** Alzheimer's disease, dementia, neurocognitive disorders, neuropsychiatry, neuropsychology

## Abstract

Early detection of Alzheimer's disease is vital for developing novel treatments. Attempts to identify the intermediate state between normal cognition and dementia have evolved over the past 50 years. Current taxonomies of mild cognitive impairment (MCI) may be criticised for their imprecise operationalisation. With the advent of biomarkers such as amyloid-beta positron emission tomography imaging in established Alzheimer's disease, much research has focused on establishing which factors predict progression from MCI to Alzheimer's disease dementia. In this review, we discuss the historical context of MCI before reviewing the literature of MCI subtypes and their risk of progression to Alzheimer's disease dementia. Finally, we summarise the literature and discuss limitations and weaknesses of how the construct is operationalised and implemented, before offering suggestions for development of the concept of MCI. We conclude that MCI must be empirically defined for the sake of its predictive validity to identify Alzheimer's disease before dementia develops.

Dementia (in this review, we will use the term dementia because of its ubiquity, the fact that it is still used by ICD-10 and historically many studies have used the term dementia), now also known as major neurocognitive disorder,^1^ is a common clinical syndrome that is characterised by progressive cognitive impairment that is severe enough significantly to impair daily functioning.^2^ Much research effort has been directed towards Alzheimer's disease, which is the most common cause of dementia.^3,4^ Despite its tremendous burden, no disease modifying treatments for Alzheimer's disease are available.^5,6^

The dominant theory of Alzheimer's disease pathophysiology implies that amyloid-beta (Aβ) is central to the upstream mechanism of disease.^7^ Recent trials with monoclonal antibodies against Aβ, such as solanezumab, have proved unsuccessful in mild to moderate Alzheimer's disease dementia^8^ and in mild Alzheimer's disease dementia,^9^ although the negative results may relate to the late disease stage at which the treatment was applied. With Aβ deposited in the brain for over 20 years before the development of the clinical syndrome of Alzheimer's disease dementia,^10^ early recognition will be key to developing potential disease-modifying therapies and secondary prevention, as well as making lifestyle and medico-legal decisions while cognitive faculties are still sufficiently intact.

Efforts to identify early or even pre-dementia patients with some very mild degree of impairment have been underway for over 50 years,^11^ and this thinking has evolved through several iterations to arrive at the current term of mild cognitive impairment (MCI).^12^ The concept of MCI has several similar but importantly different definitions and taxonomies, which will now be discussed systematically.

The review begins with a background consideration of Alzheimer's disease and an historical overview of MCI. This will be followed by a systematic review of the literature comparing the various taxonomies in their usefulness in predicting progression from MCI to Alzheimer's disease dementia. Finally, we discuss the state of the current literature and its limitations with a view to early identification of Alzheimer's disease to allow the testing of novel putative disease-modifying treatments.

## Alzheimer's disease

Alzheimer's disease is a progressive neurodegenerative condition that is the most common cause of dementia, accounting for approximately 50–70% of cases.^13–17^ Its clinical hallmark is impairment of memory and new learning with rapid forgetting of newly learned information.^18^ Diagnostic criteria emphasise impairment of memory with insidious onset and gradual progression, as well as impairment of at least one other cognitive domain, which are severe enough to impair functional abilities significantly.^1,18–21^ The most recent iteration of the DSM has adopted the term ‘major neurocognitive disorder due to Alzheimer's disease’, while retaining the essential diagnostic criteria.^1^

## Mild cognitive impairment

MCI is an intermediate state between cognitively intact persons and those with dementia. This concept has evolved over time with various taxonomies, nomenclatures and definitions, which are summarised in Table 1 and described in an historical context below.

**Table 1:** Various definitions of cognitive impairment that is not dementia

Term	BSF^11^	CDR/QD^34^	AAMI^24^	AACD^31^	CIND^41^	Petersen MCI^39^	Winblad MCI^12^	NIA-AA^43^	mNCD^1^
**Cognitive complaint**	–	–	Self- or carer- complaint about memory	Self- or carer- complaint about cognition	–	Self-complaint about memory	Self- or carer- complaint about cognition	Self- or carer- complaint about cognition	Self- or carer- complaint about cognition
**Psychometric impairment**	–	–	1 s.d. below healthy young adults	1 s.d. below age-matched sample	Battery of neuro-psychological tests	1.5 s.d. below age- and edu- matched sample	No cut-off specified.	Typically 1–1.5 s.d. below age- and edu-matched sample	Modest impairment; typically 1–2 s.d. below age- and edu- matched sample
**ADL**	–	Slight or mild change	–	–	–	Normal	Minimal impairment in complex instrumental ADL	Mild change, but still independently functioning	Independent, but possibly with greater effort or strategies
**Dementia**	No	No	No	No	No	No	No	No	No
**Notes**		Mild dementia may have CDR = 0.5		At least 6 months duration	Does not exclude non-dementia causes	AKA Mayo criteria	AKA Revised Mayo criteria or revised Petersen criteria	Biomarker criteria not presented here	

AACD, aging-associated cognitive decline; AAMI, aging-associated memory impairment; ADL, activities of daily living; AKA, also known as; BSF, benign senescent forgetfulness; CDR, Clinical Dementia Rating scale; CIND, cognitive impairment not dementia; edu, education; MCI, mild cognitive impairment; mNCD, mild neurocognitive disorder; NIA-AA, National Institute on Aging and the Alzheimer's Association; QD, questionable dementia.

### Historical development

The concept of pre-dementia causing subsyndromal symptoms was described as early as 1962, when Kral^11^ described ‘benign senescent forgetfulness’. This encompassed mild fluctuating retrieval-based memory impairment, which he speculated could be a mild early form of senile atrophy that spared the Papez^22^ circuit. A shortcoming of Kral's description was that it lacked operational criteria, which can impede diagnostic reliability.^23^

Over 20 years later, Kral's concept was extended and operationalised by Crook and colleagues; they labelled their concept age-associated memory impairment (AAMI), which they defined as subjective memory complaint and objective memory impairment on a memory test at least one standard deviation below the mean for young adults.^24^ By using healthy young adults as a reference sample, this definition lacked specificity, given that performance on psychometric tasks of memory declines with healthy ageing^25–27^ and up to 90% of elderly individuals would fulfil this criterion.^28^ A further criticism was that AAMI exclusively focused on memory, although other cognitive domains, such as visuospatial abilities, language or executive functions, may be affected principally early in Alzheimer's disease.^29,30^

Addressing both of these criticisms, the International Psychogeriatric Association broadened the concept to include other cognitive domains and also defined objective impairment with reference to an age-matched sample.^31^ They labelled this age-associated cognitive decline (AACD), defined as subjective cognitive decline as observed by the individual or an informant; gradual decline over at least 6 months; and impairment in a cognitive domain with performance one standard deviation below the mean of an age- and education-matched normative sample.^31^ AAMI and AACD appear to be distinct clinical entities with only approximately 50% overlap in concordant diagnosis and AACD participants showing more extensive cognitive impairment.^32^

The term ‘MCI’ was first described by Reisberg and colleagues with the development of the Global Deterioration Scale.^33^ This was a seven-point ordinal scale from ‘no cognitive decline’ to ‘severe dementia’ that defined MCI as one or more of several examples of cognitive lapse such as becoming lost in an unfamiliar location, word-finding difficulty, forgetting names or misplacing objects, or as concentration deficit with clinical testing.^33^

Concurrently, the clinical dementia rating (CDR) scale was developed,^34^ which was also an ordinal scale ranging from ‘no impairment’ to ‘severe dementia’. Although not directly referring to MCI, the CDR introduced the importance of daily functioning into the concept. A person scoring 0.5 or ‘questionable impairment’ on the CDR may have slight impairment of community affairs or home life but would be fully independent with self-care.^34^ Flicker and colleagues used the term ‘MCI’ when they showed that psychometric impairment at baseline could predict subsequent decline in elderly patients after 2 years.^35^

Ronald Petersen, a major developer of the concept of MCI through the Mayo clinic, developed his original definition of MCI based on patients recruited from a community-based medical clinic.^36^ They identified people who were themselves concerned about their cognition, or whose carers or physicians were concerned. These patients then had an extensive battery of physical examination, cognitive assessment, investigations and neuroimaging to rule out dementia as determined by expert panel consensus. These patients by definition had ‘normal’ scores on the Mini-Mental State Examination^37^ and Short Test of Mental Status.^38^ Petersen and colleagues (1995) observed that this cohort tended to perform 1.5 standard deviations below the age-matched mean performance on memory tasks such as auditory verbal learning tests, and activities of daily living (ADL) were generally preserved, corresponding to a CDR rating of 0.5. By employing age-corrected, but not education-corrected, normative data, it introduced confounding difficulties with patients with low education or low IQ.

These criteria were more formally proposed and became known as the Mayo Clinic core criteria or the Petersen criteria.^39^ The criteria were restricted to memory impairment rather than impairment of other cognitive domains, and thus were subject to similar criticism to that of AAMI; that Alzheimer's disease may principally affect other cognitive domains.^29^ In 2003, a key symposium of experts revised the Mayo Clinic criteria to include domains other than memory.^12^ Referred to as the Winblad criteria, these defined MCI as: (a) the person is neither normal nor demented; (b) there is evidence of cognitive deterioration shown by either objectively measured decline over time and/or subjective report of decline by self and/or informant in conjunction with objective cognitive deficits; and (3) ADL are preserved and complex instrumental functions are either intact or minimally impaired.^12^ Subcategories of MCI were established based on the pattern of cognitive domains affected: amnestic single-domain, amnestic multiple-domain, non-amnestic single-domain and non-amnestic multiple-domain.^40^

The concept of ‘cognitive impairment, no dementia’ (CIND) was introduced in the context of the need for early recognition of dementia.^41^ CIND was identified on the basis of a consensus conference of physician, nurse and neuropsychologist, integrating all available information from clinical and psychometric assessment.^42^ It includes individuals with non-dementia-related aetiologies such as delirium, chronic alcohol and drug use, depression, psychiatric illness, intellectual disability and circumscribed memory impairment; this results in high prevalence estimates^41^ and many CIND individuals will not develop dementia. A criticism of CIND is that it does not provide operational criteria, which may jeopardise its reliability.

### Recent definitions and developments

In the context of emerging biomarkers, the National Institute on Aging and the Alzheimer's Association (NIA-AA) convened a workgroup to revise the diagnostic criteria for pre-dementia Alzheimer's disease.^43^ Not long thereafter, the DSM-5^1^ abandoned the term ‘dementia’ and replaced it with ‘major neurocognitive disorder’, while adding the term ‘mild neurocognitive disorder’ (mNCD), which has similarities to MCI including cognitive complaint, psychometric impairment and relative preservation of ADL.

The NIA-AA and DSM-5 mNCD both refrained from offering a strict cut-off score for psychometric impairment, instead suggesting that typical levels of impairment would be 1–2 or 1–1.5 standard deviations below the mean, respectively, for age- and education-matched normative data. Instead of arbitrary cut-offs, these criteria advocated for an individualised assessment that incorporated all available evidence.

The NIA-AA criteria^43^ combined core clinical criteria with clinical research criteria, which incorporated biomarker evidence of disease. In doing so, these criteria moved beyond MCI as a pre-clinical definition incorporating history and examination findings to a prodromal state with biological evidence of incipient disease. The NIA-AA workgroup explicitly focused on MCI due to Alzheimer's disease and used biomarkers to stratify the likelihood that the cognitive change is due to Alzheimer's disease. Biomarkers indicating a high likelihood that MCI is due to Alzheimer's disease are an abnormal Aβ marker (e.g. positive PiB (Pittsburgh compound B) scan or cerebrospinal fluid (CSF) Aβ_42_) and a positive biomarker of neuronal injury (e.g. CSF tau, FDG-PET (Fluorodeoxyglucose Positron Emission Tomography) or structural magnetic resonance imaging). MCI unlikely to be due to Alzheimer's disease is determined when Aβ markers and markers of neuronal injury are both negative. MCI due to Alzheimer's disease with intermediate likelihood has either Aβ markers or neuronal injury markers as abnormal, while the other is untested. Recent studies suggest this taxonomy is useful in predicting Alzheimer's disease.^44,45^ However, the invasiveness, cost and availability of these biomarkers may limit their widespread implementation in clinical settings.

Subjective cognitive decline is incorporated into modern definitions of MCI.^1,12,24,31,36,43^ MCI may be preceded by a state in which the individual experiences subjective cognitive decline that is too subtle to be detected on psychometric testing.^46^ The Subjective Cognitive Decline Initiative working party have conceptualised this as a pre-MCI state on the same spectrum towards Alzheimer's disease dementia and provided definitions^46^ that have been operationalised for research purposes.^47^

### Prevalence of MCI

Since their publication, the revised Mayo clinic criteria^12^ have been commonly adopted in the literature, and studies reported in this section used these criteria unless otherwise stated.

Prospective population-based studies show that the prevalence of MCI ranges from 15 to 22% in elderly individuals.^48,49^ Prevalence increases with age, decreases with education, and is more common in males, unmarried people and carriers of the APOE-ε4 allele.^49,50^ Prospective population-based studies have estimated incidence rates of around 6% per year, although the rate in men (over 7%) was slightly higher than that in women (under 6%).^51^

### Progression of MCI to dementia

Estimates of progression rates to dementia or Alzheimer's disease dementia are important for advising patients about prognosis and have implications for conducting research in this population. Individuals with MCI have a higher risk of developing dementia compared with the general older population incidence of 1–2% per year,^52^ although estimates vary depending on the definition or subtype of MCI, study design and follow-up period.^52–55^ Earlier definitions using the Petersen amnestic-only MCI criteria estimated rates of progression to Alzheimer's disease dementia to be 10–15% per year.^52^ A randomised controlled trial reported a progression rate of 16% per year.^55^ A meta-analysis of studies using Mayo clinic criteria for MCI suggested that over 10 years, 33.6% will cumulatively progress to Alzheimer's disease dementia in specialist settings versus 28.9% in population settings, which translated to an annual progression rate of 8.1% in specialist settings and 6.8% in community studies.^56^

There is some criticism of the utility of MCI as a diagnosis given its heterogenous nosology,^57^ variable prognostic significance^58–60^ and the various ethical issues it raises.^57^ We would counterargue that these issue provide impetus to refine the definition of MCI, as doing so will allow identification of a group that could be identified for treatment of modifiable risk factors that may decrease the risk of developing dementia, such as diet, diabetes mellitus, hypertension and hypercholesterolemia.^61,62^

The present study aimed to review the evidence with regards to which taxonomy of MCI was more useful in predicting incident Alzheimer's disease dementia. We hypothesised that amnestic MCI (aMCI) and multiple-domain MCI would be more likely than non-MCI controls to progress to Alzheimer's disease dementia.

## Methods

### Search method

**Fig. 1:**
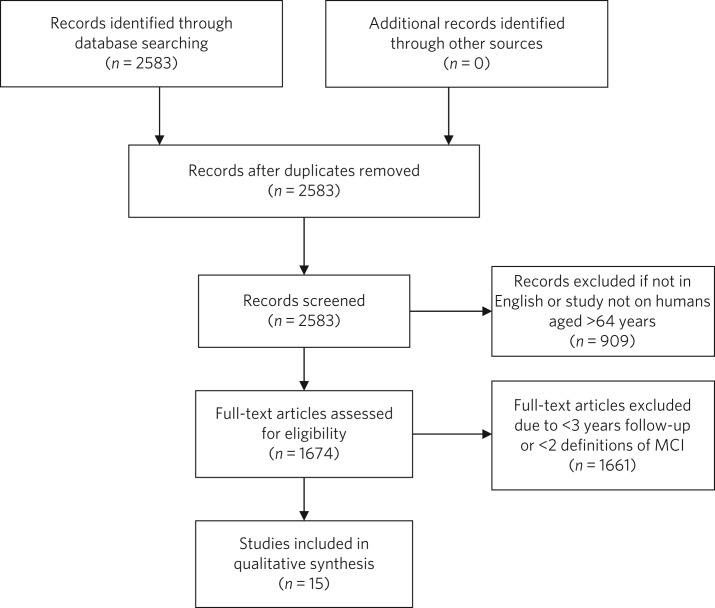
PRISMA diagram of study selection.

Medline was searched via PubMed on 28 February 2017 using the search terms ‘MCI or Mild Cognitive Impairment’ and ‘Alzheimer's disease’ and ‘progression or conversion’, identifying 2583 studies. The search was restricted to articles in the English language and studies conducted on humans aged 65 years and over, resulting in 1674 studies. See Fig. 1 for the PRISMA diagram.^63^

### Selection criteria

Studies were selected if they performed longitudinal follow-up of at least 3 years, reported on the incident development of Alzheimer's disease dementia using established criteria, and explicitly compared two definitions of MCI. The 3-year duration was selected because of the lower specificity associated with shorter follow-up.^64^

### Data extraction

All titles were reviewed and the abstracts of all potentially relevant studies were assessed. The identified full papers were assessed for eligibility and data were extracted. Study quality was assessed using the Newcastle-Ottawa Quality Assessment Scale.^65^

## Results

There were 15 studies included in the final analysis, all of which were classified as ‘good’ according to the Newcastle-Ottawa Quality Assessment Scale.^65^

### MCI subtype and progression to Alzheimer's disease dementia

Only a single study explicitly examined differences between various classification systems of MCI and progression to Alzheimer's disease dementia. In a large population-based study of 4057 individuals with 4.5 years follow-up, DSM-5 criteria gave a higher annual progression rate than Petersen criteria for progression to Alzheimer's disease dementia and to all-cause dementia.^66^ However, the majority of people who developed Alzheimer's disease dementia were classified as normal controls at baseline. The DSM-5 criteria were more restrictive, with only 139 cases meeting criteria, whereas 303 cases met criteria for Petersen aMCI. The authors do not stipulate why, but a possible contributing factor to this is that the DSM-5 criteria explicitly exclude people with severe depression, psychosis or delirium, whereas the Petersen criteria do not. Marcos and colleagues (2016) noted that most of the MCI cases did not progress to Alzheimer's disease dementia or dementia during the 4.5-year follow-up; indeed, only 15% of the DSM-5 defined MCI cases progressed to dementia.

Twelve studies explicitly examined differences between various subtypes of MCI, usually within the Winblad taxonomy.^12^ The most consistent finding was that aMCI is associated with an increased risk of progression to Alzheimer's disease dementia.^53,67–77^ Individuals with aMCI are more likely (18–19% per year) to progress to Alzheimer's disease dementia than non-amnestic MCI participants (10–11%) in community-^53^ and healthcare-based cohorts.^78^

Ten studies compared progression rates between various subtypes within the Winblad taxonomy. Seven of these studies show that multiple-domain aMCI has the best predictive accuracy for progression to Alzheimer's disease dementia,^54,67–69,73,74,76^ with annual progression rates ranging from 4 to 25%. However, two studies found that single-domain aMCI was associated with the highest risk of progression to dementia due to Alzheimer's disease,^70,77^ and one found no difference between single- and multiple-domain aMCI.^71^ A challenge to the discriminative validity of the Winblad taxonomy is that multiple-domain aMCI was also the best predictor of progression to vascular dementia.^73^

Although all studies purported to employ the revised Mayo criteria, these were operationalised in different ways, for example, using hierarchical cluster analysis of neuropsychological data rather than clinical judgement^70^ or not including information about subjective memory complaint.^67^ Moreover, psychometric impairment was defined in one study as at least 1.5 standard deviations below the mean for an age- and education-matched sample on a neuropsychological battery^77^ or as at least 1.0 standard deviations below the mean for an age- and education-matched sample on indices derived from the Montreal Cognitive Assessment.^68^

## Discussion

The concept of MCI has evolved from a vague clinical observation to a diagnosis that can incorporate disease biomarkers to predict the likelihood of developing Alzheimer's disease dementia. There have been at least nine different attempts to define the intermediate state between cognitive health and dementia. However, only a single study has explicitly compared different taxonomies in terms of their usefulness in predicting incident Alzheimer's disease dementia.^66^ This study showed that DSM-5-defined mNCD had better positive predictive value than did Petersen criteria, although the majority of people who developed Alzheimer's disease dementia were classified as normal controls at baseline. Of the studies comparing various subtypes of MCI within the Winblad taxonomy, aMCI better predicts progression to Alzheimer's disease dementia than does non-amnestic MCI.^53,78^ This is consistent with the observation that memory impairment is the hallmark clinical feature of Alzheimer's disease.^18^

Although there was not consensus, 7 of 10 studies found that multiple-domain aMCI was better than single domain aMCI in predicting progression,^54,67–69,73,74,76^ two showed the opposite^70,77^ and one showed no difference.^71^ A possible reason for the discrepant findings regarding single-domain aMCI and multiple-domain aMCI in the prediction of Alzheimer's disease dementia is differing definitions of the subtypes. The inconsistent findings within this area highlight the variable implementation of the criteria. Although all studies purported to employ the revised Mayo criteria, these were operationalised in different ways, such as not including subjective memory complaint,^67^ different psychometric cut-off *z-*scores ranging from −1.0^68^ to −1.5,^77^ different psychometric tests^68,77^ or even hierarchical cluster analysis of neuropsychological data.^70^

This review suggests that aMCI is superior to non-amnestic MCI and that multiple domain aMCI is probably superior to single domain aMCI in predicting progression to Alzheimer's disease dementia. It may be that involvement of cognitive domains in addition to memory in MCI implies more severe or advanced disease that is closer to the emergence of dementia. Despite these findings, the predictive validity of MCI is limited, as up to 60% of MCI individuals will not develop dementia in the following 10 years.^56^

We suggest that the concept of MCI may be improved in three ways. First, criteria should be operationally defined. Second, criteria should be empirically defined. Finally, the MCI group should be stratified for likelihood of progression to Alzheimer's disease dementia. These will now be discussed in turn.

Several taxonomies of MCI have suggested explicit cut-off scores on cognitive measures. Despite this, more recent taxonomies from the DSM-V and NIA-AA have dispensed with cut-offs for cognitive impairment. Although this approach has the merit of tailoring assessment to the individual, it may introduce issues with interrater reliability, which may further undermine the reliability of MCI in the research literature. We suggest that criteria for subjective and objective memory impairment should be operationalised to ensure reliability of the concept.

This raises the question of which cut-off should be adopted. We suggest that the utility of MCI may be improved by providing operational criteria that are empirically defined by their prediction of Alzheimer's disease dementia. There have been only a few attempts to use such data-driven definitions of MCI. For example, MCI subtypes identified with latent profile analysis outperformed Winblad criteria^79^. Other studies have shown that the severity of memory impairment^80,81^ and the base rate of memory impairment^82^ offer an advantage over the common taxonomies. We propose that cognitive impairment used to identify MCI should be empirically defined, whether it be in terms of the lowest performance,^80,81^ base rate of impairment,^82^ or possibly average memory score or some other method.

These same factors may then be used to stratify the severity or grade of MCI. All current taxonomies treat MCI as a categorical entity, which is not consistent with a longitudinal model of Alzheimer's disease pathophysiology. Although the clinical manifestation of Alzheimer's disease exists on a spectrum from asymptomatic to severe dementia, MCI is not staged as such. We propose that MCI should be stratified by factors such as severity^80,81^ or base rate of impairment^82^ to indicate increased risk of progression to Alzheimer's disease dementia. This may allow individuals to be selected for more intensive monitoring, for secondary prevention techniques such as control of diet and cardiovascular risk factors,^61,62^ and for recruitment into clinical trials of putative treatments for Alzheimer's disease.

## Conclusion

The current literature suggests that MCI individuals with memory impairment and impairment of multiple domains are at increased risk of progression to Alzheimer's disease dementia. We suggest that the concept of MCI should be improved by offering operational criteria of memory or cognitive impairment that are empirically defined. Furthermore, we propose that MCI should be developed from a singular categorical diagnosis to a graded diagnosis that indicates increased risk for progression to Alzheimer's disease dementia. In this way, MCI may become a more reliable construct with better predictive validity that will be more useful in understanding the natural history of Alzheimer's disease. This in turn will allow better targeted selection of individuals with pre-symptomatic Alzheimer's disease to allow early implementation of therapeutic strategies to modify the course of this common and burdensome disease.

## About the authors

**Nicholas I. Bradfield** is a basic physician trainee at St Vincent's Hospital, Melbourne, Australia and is a clinical neuropsychologist in private practice in Melbourne, Australia. **David Ames** is Emeritus Professor in the University of Melbourne Academic Unit for Psychiatry of Old Age, St George's Hospital, Kew, Australia, a consultant psychiatrist with Melbourne Heath, Parkville, Australia, and director of the National Ageing Research Institute, Parkville, Australia.
